# Normative data and clinically significant effect sizes for single-item numerical linear analogue self-assessment (LASA) scales

**DOI:** 10.1186/s12955-014-0187-z

**Published:** 2014-12-18

**Authors:** Jasvinder A Singh, Daniel Satele, Suneetha Pattabasavaiah, Jan C Buckner, Jeff A Sloan

**Affiliations:** Department of Medicine, University of Alabama at Birmingham, Birmingham, AL USA; Medicine Service, Veterans Affairs Medical Center, Birmingham, AL USA; Department of Orthopaedic Surgery, Mayo Clinic, Rochester, MN USA; Department of Psychiatry, Moffitt Cancer Center, Tampa, FL USA; Department of Health Sciences Research, 200 First Street SW, Rochester, MN 55905 USA; Department of Medical Oncology, Mayo Clinic, Rochester, MN USA

**Keywords:** Quality of life, Measurement, LASA, Validation, Single item, Linear analog scale, Patient-reported outcomes, PROs, QOL

## Abstract

**Background:**

Single-item assessments have been the most often-used measures in National Cancer Institute (NCI) cancer control clinical trials, but normative data are not available. Our objective was to examine the normative data and clinically significant effect sizes for single-item numerical linear analogue self-assessment (LASA) scale for overall quality of life (QOL).

**Methods:**

We analyzed baseline data from 36 clinical trials and 6 observational studies with various populations, including healthy volunteers, cancer trial patients (patients with advanced incurable cancer or patients receiving treatment with curative intent) and hospice patients as well as their caregivers. The overall QOL LASA was rated 0 (as bad as it can be) to 10 (as good as it can be). We calculated the summary statistics and the proportion of patients reporting a clinically meaningful deficit (CMD) of a score equal to 5 or less on the 0–10 scale.

**Results:**

In total, for the collective sample of 9,295 individuals, the average overall QOL reported was 7.39 (SD = 2.11) with a markedly skewed distribution with roughly 17% reporting a score of 5 or below indicating a clinically significant deficit in overall QOL. Hospice patients report a much worse average score of 5.7 upon entry to hospice; hospice caregivers average 7.4. Cancer patients vary within these two extremes with most patients averaging in the 7’s on the 0–10 scale (range, 0 to 10 p-value < 0.0001). Men and women’s QOL distributions were virtually identical (with average of 7.6 vs. 7.5, p-value = 0.046). Overall QOL was weakly related to performance status with a Spearman correlation coefficient of −0.29 (p-value < 0.0001). Overall QOL was related to tumor response (p-value = 0.0094), i.e. patients with a full or partial response reported a CMD in 11.4% of cases compared to 14.4% among those with stable disease and 18.5% among those with disease progression. Data missingness was high for performance status and tumor response associations.

**Conclusions:**

This study provides the normative data for cancer patients and healthy volunteers for overall QOL using the LASA. These can serve as benchmarks for future studies and inform clinical practice decision-making.

**Electronic supplementary material:**

The online version of this article (doi:10.1186/s12955-014-0187-z) contains supplementary material, which is available to authorized users.

## Background

Patient-reported outcomes (PROs) are increasingly becoming the focus of research and clinical practice [1,2]. A major challenge in the use of PROs is the practical consideration of the number of items that can be asked [3]. Considerable evidence has been generated to demonstrate the value of simple single-item PRO assessments for describing the effects of disease and treatment in cancer and other diseases [4–6]. Single-item assessments in fact have been the most often-used measures in National Cancer Institute (NCI) cancer control clinical trials [7,8]. The purpose of this manuscript was to present normative data for a specific set of single item PRO measures that have been used in numerous clinical trials and clinical practice settings so as to serve as a reference resource.

Linear Analogue Self Assessment (LASA) items have been validated as general measures of global QOL dimensional constructs in numerous settings [9–13]. The acronym LASA actually only refers to the type of response scale, but has come to be associated with simple single-item PRO measures in clinical research. This is partially due to the wide application of these simple measures and the ready acceptance by clinical researchers and clinicians. These single-item assessments have become the most-used assessment in all NCI-sponsored cancer control studies [8]. Single-item tools are in widespread use, for example JCAHO has mandated that single-item pain assessments be completed at the time of every clinical intake for institutions to maintain accreditation [14]. The incorporation of these requirements into clinical practice presumes patient care has improved although the evidence is inconsistent [15]. Recently, a PRO Outcome Measurement System (PROMIS) paper compared a single-item pain measure to a longer assessment and indicated that the two were psychometrically similar but complimentary [16]. This would seem to indicate that there is a place for both in the clinical trials armamentarium.

The advantages of the LASA include brevity and minimized burden for both the patient and the clinical or research system. Sloan and colleagues explored the advantages and disadvantages of single versus multiple item approaches extensively [1] and have further demonstrated that where an indication of clinically significant deficits is the goal of assessment, the LASAs are superior to longer multi-item scales. This is in part due to the LASA allowing a patient to make the gestalt combination for sub-constructs rather than a predetermined metric formula derived empirically from a factor analysis for example [3]. Trusting that the patient has this capability is a key assumption to success. Whatever a patient says their QOL is, that is what it is. Some psychometric analyses assume that the patient has to be fooled into providing an accurate score or that they may give an “inaccurate score”. This is condescending and paternalistic in the extreme. As a triage screening or trigger, an individual LASA has the most obvious application. Also, the brevity allows for routine application in clinical settings where a longer tool would be economically and temporally prohibitive.

The disadvantages of the LASA include a lack of detail about the deficit indicated by the single item. Others have pointed to a lack of capability to obtain a measure of reliability for a single item. However, as demonstrated by Cleeland, if the construct being measured is valid and understandable to the subject, then unidimensional reliability is automatically present [17,18]. Furthermore, recent research has indicated that reliability for single-items can indeed be measured by using correction for attenuation or factor analysis [19].

The LASA have become the focus of a specific line of research into prognostic factors for survival. Specifically, single item measures of overall QOL and fatigue have been seen to be prognostic for survival in multiple disease groups [1,20–22].

Typically, LASAs are scored on a 0 to 10 scale. Initially a true linear analogue (i.e. a line) was presented to patients who were asked to then place an X on the line to represent their score. This had the benefit of producing a “continuous” variable, but was arduous in terms of scoring, as staff would have to use a ruler to measure the score on each item. Research indicated that patients tended to clump around the middle and quartiles of the line so that the true measurement accuracy that was being provided was realistically a five-point scale with errors around each point. Subsequent LASAs hence used a 0–4 or 0–10 numerical response scale (NRS). In some papers one will see NRS instead of LASA for a label to be psychometrically precise. The use of the 0–10 scale was demonstrated by Norman et al. to have advantages over other alternatives, although 0–4, 1–7, 1–5 and other response scales have all been employed [23]. A linear transformation of any such scaling can be used to translate all scores onto a 0–100 point scale.

A score 50 or below on the transformed 0–100 scale is indicative of a need for immediate exploration and intervention for the QOL deficit [4,24]. Due to these findings, the NCCTG and subsequently the Alliance for Clinical Trials in Oncology (Alliance) decided to include LASA measures for overall QOL and fatigue in all future phase II and phase III clinical trials as an independent prognostic factor independent of performance status. Our study objective was to analyze and present normative data for LASA measures from various patient and control populations.

## Methods

This paper presents a series of normative data for overall QOL LASA scale (Additional file 1) drawn from different populations ranging from healthy volunteers to hospice patients. In total, baseline QOL LASA data from 36 clinical trials and 6 observational studies are included (Table 1). The reference indicated for each study was either a published manuscript, a protocol, an abstract or unpublished dataset as indicated. Healthy NCCTG volunteers (54) provided LASA data via a survey at a semi-annual meeting. Mayo physician and residents data is drawn from a survey. Please refer to Additional file 2 for the details about where each sample was obtained.

**Table 1:** **Data sources, population type, summary statistics for overall QOL**

**Patient category**	**N**	**Mean**	**Median**	**Minimum**	**Maximum**	**Standard deviation**
Advanced cancer	120	7.26	8.10	0.20	9.70	2.31
Brain cancer	26	6.46	7.00	2.00	9.00	1.88
Breast cancer	296	7.77	8.20	1.40	10.00	1.84
Lung cancer	1155	7.25	7.80	0.00	10.00	2.10
Colon cancer −2 Wks Post Surgery (Colectomy)	388	7.57	8.00	1.50	10.00	1.80
Colon cancer-Pre Surgery (Colectomy)	403	8.06	8.50	2.00	10.00	1.75
GI cancer	2409	7.74	8.30	0.00	10.00	1.86
GU cancer	180	8.29	9.00	2.40	10.00	1.61
Gynecologic cancer	117	7.83	8.20	1.80	10.00	1.75
Head and neck cancer	254	7.24	7.80	0.00	10.00	2.27
Hematologic cancer	32	7.33	7.70	1.70	9.30	2.03
Lymphatic cancer	8	7.21	8.45	3.20	9.20	2.51
Multiple site cancer	14	8.02	8.00	6.00	10.00	1.56
Musculoskeletal site cancer	18	6.68	7.55	1.80	10.00	2.78
Neurologic cancer	214	7.42	8.00	1.00	10.00	1.90
Other cancer	52	7.91	8.80	1.00	10.00	2.34
Lung cancer- Mayo study	529	7.03	7.00	0.00	10.00	2.18
Lung cancer - Mayo study 6 months post diagnosis	1409	7.05	7.60	0.00	10.00	2.40
Skin cancer	7	7.59	7.90	5.90	9.40	1.17
Unknown site cancer	29	6.60	7.00	2.10	10.00	2.25
Healthy NCCTG volunteers	54	8.31	9.00	5.00	10.00	1.19
Hospice caregivers	53	7.44	7.50	3.75	10.00	1.74
Hospice	52	5.89	5.75	2.00	9.75	2.03
Minnesota medical students	543	7.16	7.00	1.00	10.00	1.76
Mayo physicians	460	7.30	7.00	1.00	10.00	1.69
Mayo residents	295	6.46	7.00	1.00	10.00	1.91

GI, gastroenterological; GU, genitourinary; NCCTG, North central chapter treatment group.

Distributions for the various cohorts of Table 1 are displayed in Figures 2 and 3.

Simple summary statistics (means, standard deviations) are the primary analytical tool for this work. Correlation between the LASA and other measures/demographics was accomplished via correlation coefficients. We compared LASA scores across subpopulations by Fisher’s exact tests for categorical variables and Kruskal-Wallis testing for continuous variables.

## Results

**Figure 1:**
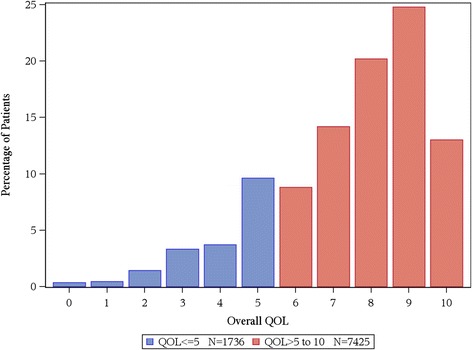
**QOL scores were not available for 134 patients.** Distribution of overall QOL score, N=9,161.

**Figure 2:**
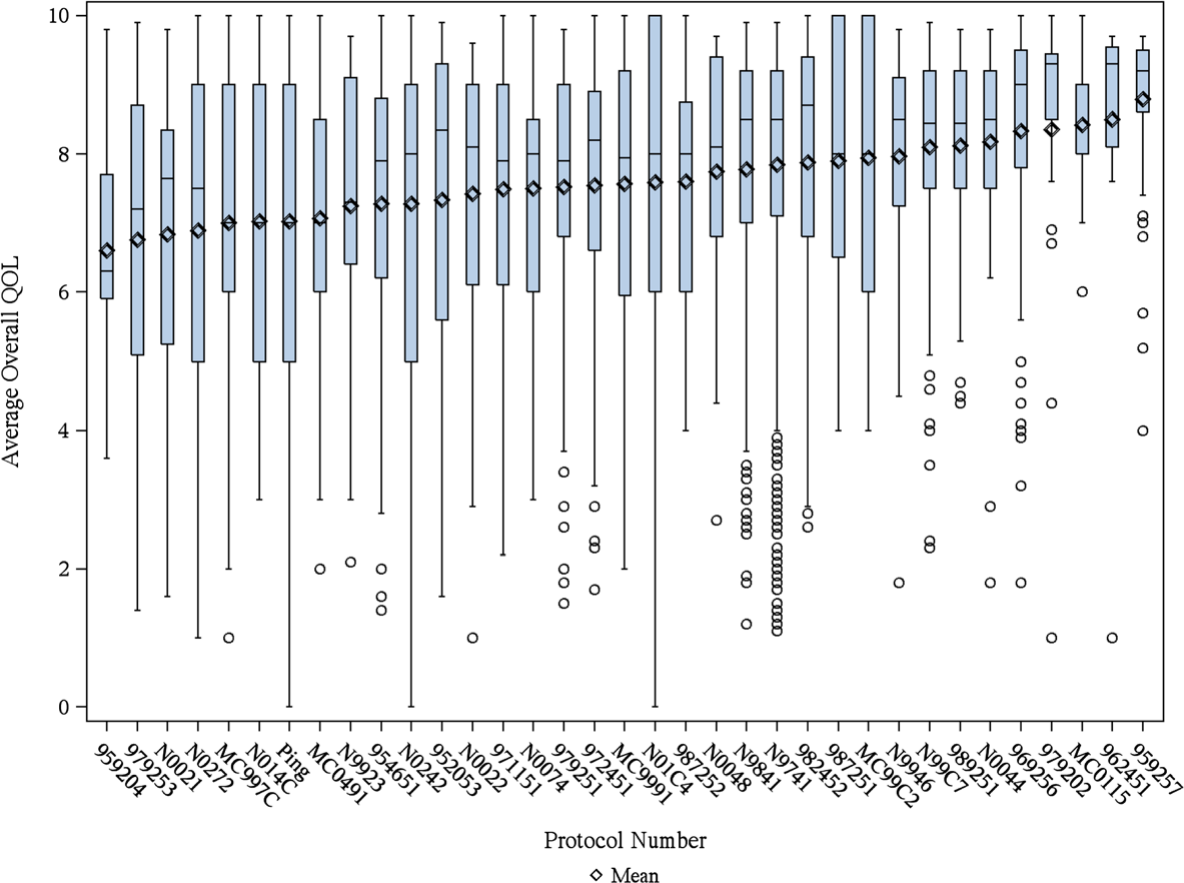
**Error bars indicate standard deviation; medians are indicated by the horizontal lines with in each graphic.** Boxplots of Overall QOL for individual studies.

**Figure 3:**
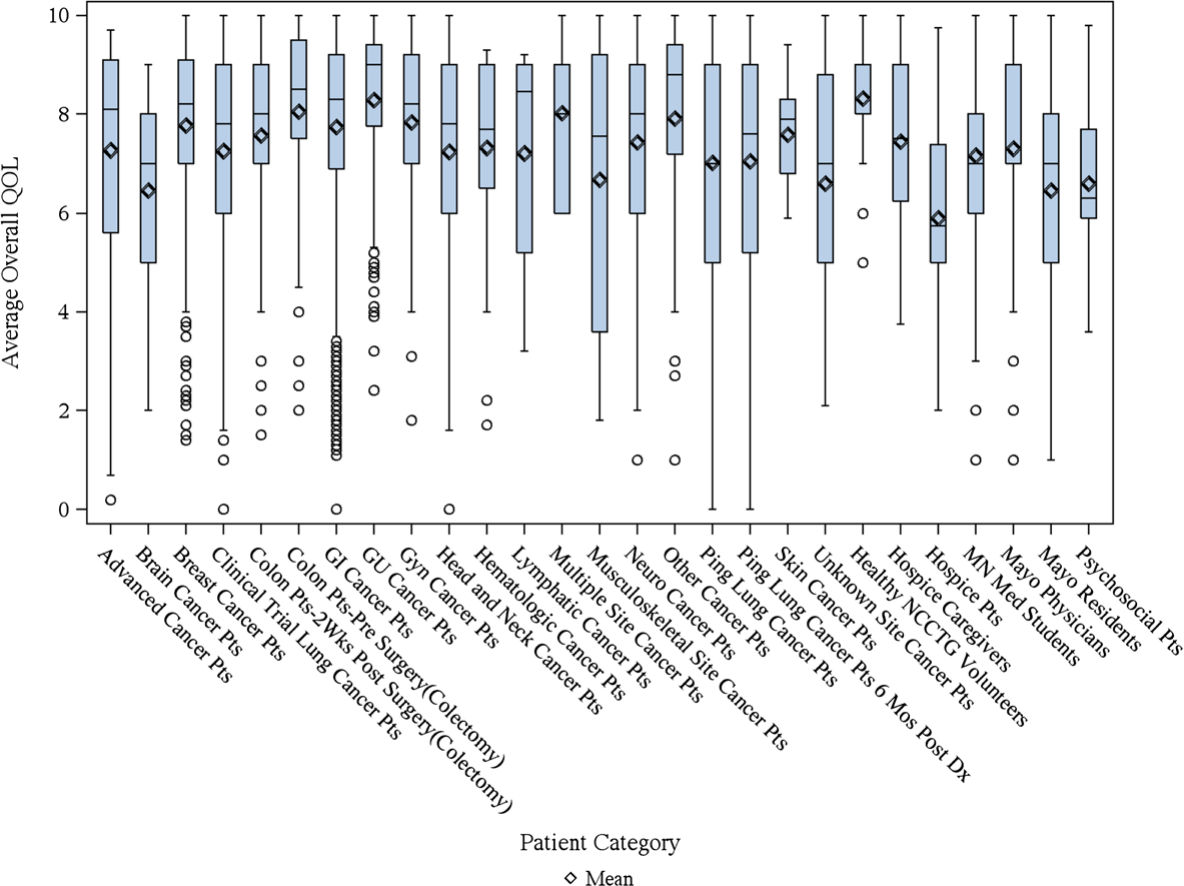
**Error bars indicate standard deviation; medians are indicated by the horizontal lines with in each graphic.** Boxplots of Overall QOL for Patient Categories.

**Figure 4:**
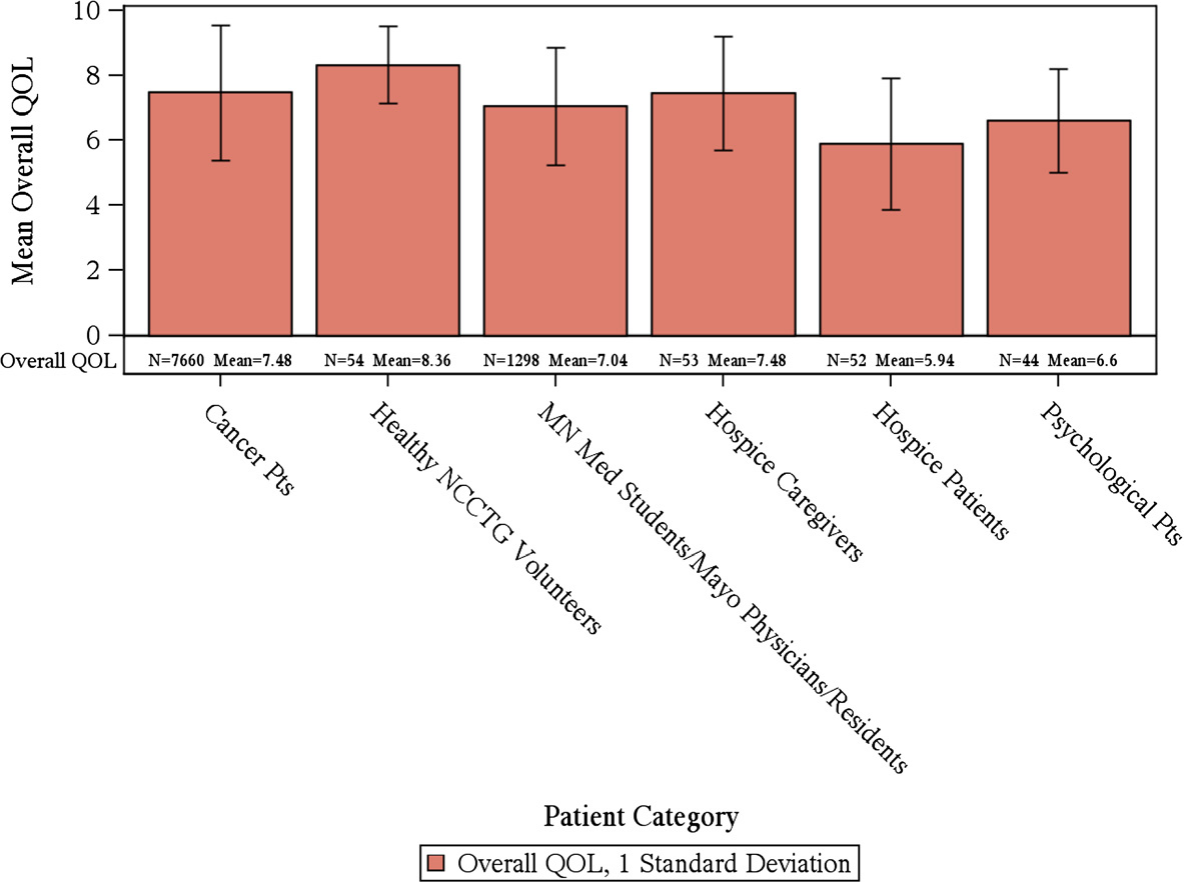
**Error bars indicate standard deviation.** Mean Overall QOL scores.

In total, for the collective sample of 9,295 individuals, the average overall QOL reported was 7.39 (SD = 2.11) with an overall distribution displayed in Figure 1. The distribution is markedly skewed with roughly 17% reporting a score of 5 or below indicating a clinically significant deficit in overall QOL. Distributions for the various cohorts of Table 1 are displayed in Figures 2 and 3. Comparison of overall QOL scores for select groups is shown in Figure 4.

Healthy individuals average above 8.3 (SD = 1.2) on the 0–10 scale and rarely report a score of 5 or below indicating a clinical deficit (Tables 1 and 2). Hospice patients report a much worse average score of 5.7 upon entry to hospice, although it has been seen that their QOL will improve after hospice care has been initiated [25]. Hospice caregivers average 7.4. Cancer patients vary within these two extremes with most patients averaging in the 7’s on the 0–10 scale. Health care professionals score on average almost as bad as their patients. In particular, Mayo Physicians and Minnesota Medical students average 7.3 while residents averaged 6.5. The full range of the scale was reported by almost all cohorts except for healthy individuals, hospice caregivers and skin cancer patients.

Table 2 differentiates patient cohorts by the proportion of patients reporting a clinically meaningful deficit (CMD) of a score equal to 5 or less. This CMD is related to a relative doubling for the risk of death [1]. Healthy volunteers rarely (2%) reported a clinically significant deficit. Hospice patients upon entry had the highest prevalence (42%) of clinically significant deficits in overall QOL.

**Table 2:** **Incidence of clinically significant deficits in QOL for each cohort**

**Cohorts**		**QOL score on 0–10 scale N (%)**			
	**Missing**	**<=5**	**>5 to <8**	**8-10**	**Total**
Advanced cancer	7	25 (21%)	30 (25%)	65 (54%)	120
Brain cancer	0	8 (31%)	11 (42%)	7 (27%)	26
Breast cancer	0	27 (9%)	94 (32%)	175 (59%)	296
Clinical trial lung cancer	0	236 (20%)	350 (30%)	569 (49%)	1155
Colon cancer pts-2 wks post surgery (Colectomy)	63	67 (17%)	118 (30%)	203 (52%)	388
Colon cancer pts-pre surgery (Colectomy)	48	58 (14%)	85 (21%)	260 (65%)	403
GI cancer	0	287 (12%)	682 (28%)	1440 (60%)	2409
GU cancer	0	14 (8%)	41 (23%)	125 (69%)	180
Gynecologic cancer	0	14 (12%)	35 (30%)	68 (58%)	117
Head and neck cancer	0	54 (21%)	78 (31%)	122 (48%)	254
Hematologic cancer	0	4 (13%)	13 (41%)	15 (47%)	32
Lymphatic cancer	0	2 (25%)	1 (13%)	5 (63%)	8
Multiple site cancer	0	0 (0%)	5 (36%)	9 (64%)	14
Musculoskeletal site cancer	0	6 (33%)	5 (28%)	7 (39%)	18
Neurologic cancer	0	25 (12%)	75 (35%)	114 (53%)	214
Other cancer	0	8 (15%)	8 (15%)	36 (69%)	52
Lung cancer- Mayo study	0	137 (26%)	143 (27%)	249 (47%)	529
Lung cancer- Mayo study 6 months post diagnosis	0	326 (23%)	426 (30%)	657 (47%)	1409
Skin cancer	0	0 (0%)	4 (57%)	3 (43%)	7
Unknown site cancer	0	10 (34%)	9 (31%)	10 (34%)	29
Healthy NCCTG volunteers	0	1 (2%)	12 (22%)	41 (76%)	54
Hospice caregivers	4	9 (17%)	23 (43%)	21 (40%)	53
Hospice	5	22 (42%)	23 (44%)	7 (13%)	52
Minnesota medical students	2	84 (15%)	202 (37%)	257 (47%)	543
Mayo physicians	5	58 (13%)	174 (38%)	228 (50%)	460
Mayo residents	0	83 (28%)	125 (42%)	87 (29%)	295
Advanced cancer –psychosocial study	0	10 (23%)	24 (55%)	10 (23%)	44

NCCTG, North central chapter treatment group.

In terms of cancer site, lung, brain, musculoskeletal, metastatic cancer, head and neck and lymphatic cancers had between 20-33% with CMD in overall QOL. All other cancer cohorts reported lower incidence rates of CMD in overall QOL. It was notable that 28% of Mayo residents, 13% of Mayo physicians and 15% of MN medical students reported CMDs in QOL.

Overall QOL scores were subsequently analyzed by selected demographics. Overall QOL on average declined slightly with increased age, but only one seventh of a standard deviation or a 7% increase in the percentage reporting a CMD in overall QOL (Table 3). Men and women’s overall QOL distributions were virtually identical (Table 4). When examining data separately from cancer treatment trials vs. observational studies, differences were noted by age in treatment trials, but not observational studies (Additional file 3). Most data for gender came from cancer treatment trials, which showed identical scores in men and women (Additional file 3); few data from observational studies were available that showed some gender differences (Additional file 4).

**Table 3:** **Overall QOL by age**

	**Missing (N = 2898)**	**<50 (N = 910)**	**50-64 (N = 2378)**	**65-71 (N = 1444)**	**72+ (N = 1665)**	**Total (N = 9295)**	**p value**
**Overall QOL**							0.0013^1^
N	2884	901	2350	1410	1616	6277	
Mean (SD)	7.1 (2.1)	7.5 (1.9)	7.6 (1.9)	7.6 (2.0)	7.4 (2.1)	7.5 (2.0)	
Median	7.2	8.0	8.0	8.0	8.0	8.0	
Q1, Q3	6.0, 9.0	6.6, 9.0	6.8, 9.1	6.4, 9.1	6.0, 9.0	6.3, 9.0	
Range	(0.0-10.0)	(1.1-10.0)	(0.0-10.0)	(0.0-10.0)	(0.0-10.0)	(0.0-10.0)	

^1^Kruskal Wallis test.

**Table 4:** **Overall QOL by gender**

	**Missing (N = 2931)**	**F (N = 2835)**	**M (N = 3529)**	**Total (N = 9295)**	**p value**
Overall QOL					0.25^1^
N	2906	2772	3483	6255	
Mean (SD)	7.1 (2.1)	7.6 (1.9)	7.5 (2.0)	7.5 (2.0)	
Median	7.1	8.0	8.0	8.0	
Q1, Q3	6.0, 9.0	6.6, 9.0	6.0, 9.0	6.3, 9.0	
Range	(0.0-10.0)	(0.0-10.0)	(0.0-10.0)	(0.0-10.0)	

^1^Kruskal Wallis test.

Overall QOL was weakly related to performance status with a Spearman correlation coefficient of −0.29 indicating that people with lower performance status tended to have worse overall QOL (Table 5). Roughly 14% of patients with performance status 0 or 1 reported a CMD compared to 58% reporting a CMD among patients with a performance status 2 or worse.

**Table 5:** **Overall QOL by performance score**

	**Missing (N = 5665)**	**0 (N = 1492)**	**1 (N = 1934)**	**2-3 (N = 204)**	**Total (N = 9295)**	**p value**
**Overall QOL**						<0.0001^1^
N	5531	1492	1934	204	3630	
Mean (SD)	7.3 (2.1)	8.1 (1.7)	7.4 (2.0)	6.2 (2.2)	7.6 (1.9)	
Median	7.9	8.6	8.0	6.0	8.1	
Q1, Q3	6.0, 9.0	7.4, 9.2	6.1, 9.0	4.8, 8.0	6.6, 9.1	
Range	(0.0-10.0)	(0.0-10.0)	(0.0-10.0)	(1.0-10.0)	(0.0-10.0)	

^1^Kruskal Wallis test.

Overall baseline QOL was somewhat related to subsequent tumor response (Table 6; p = 0.0094). Patients with a full or partial response reported a CMD at baseline in 11.4% of cases compared to 14.4% among those with stable disease and 18.5% among those with tumor progression.

**Table 6:** **Overall QOL by best response**

	**Missing (N = 6803)**	**Complete response (N = 140)**	**No evidence of disease (N = 14)**	**Progressive disease (N = 492)**	**Partial response (N = 709)**	**Criteria for regression (N = 115)**	**Stable disease (N = 1022)**	**Total (N = 9295)**	**p value**
**Overall QOL**									0.0094^1^
N	6669	140	14	492	709	115	1022	2492	
Mean (SD)	7.3 (2.1)	7.9 (1.6)	8.3 (1.4)	7.5 (2.0)	7.9 (1.8)	7.8 (1.9)	7.7 (1.8)	7.8 (1.9)	
Median	7.9	8.5	8.6	8.0	8.5	8.5	8.3	8.4	
Q1, Q3	6.0, 9.0	7.3, 9.1	8.0, 9.2	6.1, 9.0	7.1, 9.2	7.2, 9.2	7.0, 9.2	6.9, 9.2	
Range	(0.0-10.0)	(2.4-9.9)	(4.5-10.0)	(1.0-10.0)	(0.0-10.0)	(1.6-10.0)	(1.2-10.0)	(0.0-10.0)	

^1^Kruskal Wallis.

## Discussion

This paper provides a series of normative data drawn from multiple sources for the simple single-item measure of overall QOL that has been used in numerous clinical trials, observational research and clinical practice settings. The overall QOL item differentiates across healthy populations and various patient populations in terms of average values and the incidence of CSDs reported.

A key finding is that overall QOL is different from performance status. This result has been demonstrated previously in individual studies [3,4,26], but was demonstrated here to be consistent across study populations. Similarly, the relationship between tumor response in cancer patients and QOL is weak, as reported previously in a study of 989 metastatic colorectal cancer [27]. For example, neither baseline QOL nor changes in QOL indicated a relationship of any strength with tumor response [27]. A limitation of our analyses of associations of QOL with performance status and tumor response there was that large amount of data were missing. The impact of data missingness on our results is unclear and this must be taken into account while interpreting these results. Gender differences in reporting QOL are also nonexistent. Overall QOL also does not automatically decline with age although a general trend is present. Collectively these findings indicate that a patient’s self-reported QOL is more than merely a function of performance status, age, gender or any other demographic/clinical variable.

There are numerous existing well validated and reliable, but much longer, measures of quality of life in cancer patients, there is an overriding need for simple single item assessment measures, such as the LASA used for recording overall QOL in this study [28]. This brief QOL measure is advantageous because it reduces patient burden, both in clinical situations and in clinical trials, and has greater clinical utility for the busy practitioner [29]. Nearly 10 years ago, editors of health quality and life outcomes indicated that there may be too many QOL assessment tools, making the goal of finding an optimal tool difficult [30], as suggested from our work published in 1998 [31]. In the text book by Fayers and Machin, section 2.5 states “the simplest and most overtly sensible approach to measure QOL is to use global rating scales” “A global single item measure may be a more valid measure of the concept of interest than a score from a multi-item scale” [32]. In a series of studies, Zimmerman et al. had almost 2,000 psychiatric outpatients complete single-item assessments of psychosocial functioning and QOL, as well as more complex measures [33]. The single item measures of symptom severity, psychosocial functioning and QOL were strongly correlated with the multi-item measures and were able to discriminate among various clinical populations, e.g., depressed and non-depressed patients. They concluded that single-item measures could be easily incorporated into a busy clinical practice and were reliable and valid in order to collect data on patient condition and treatment effective. Similar results were found by Yohannes et al. in patients with cystic fibrosis [34]. Krause et al. discussed the practical utility of single-item assessments [35]. In fact, this measure is presently being used routinely in our clinical practice for every patient visit.

Results of a survey of usage of the overall QOL item indicate that it allows for clinicians to identify otherwise patient concerns and to facilitate conversations regarding the precise nature of the issues underlying the concerns [36]. A single item can play a central role in triaging and routine screening for issues that patients want addressed but that have either not been raised by the clinician or volunteered by the patient for various reasons including lack of time in the clinical visit or discomfort surrounding sensitive issues like sexuality [37].

The clinical importance of the single item overall QOL is inherent in its ability to tap into the simple construct of overall well being within a patient using his/her own internal weighting scheme for the innumerable component constructs [3]. While some multiple item measures may look at many aspects of QOL, it is impossible to cover all facets of QOL or give them appropriate weighting. Indeed, it has been previously demonstrated that a patient may report a deficit in overall QOL due to a deficit in a single sub-domain that they consider of primary importance that overrides positive indications on all other domains [1,4]. It is this gestalt capability of a single item that is likely the reason that it has been seem to be empirically linked to overall survival. In its simplest form, the item is asking “Do you think you are doing well?” This even in the presence of overwhelmingly positive objective laboratory and clinical data may be the overriding determinant of the individual’s well being. In one way, this general item can capture unknown important aspects of well being that are being the capability of presently available clinical measures.

A major drawback and concern with the use of a simple single-item measure of QOL is the lack of detail and precise determination of what is being measured or meant by “overall QOL” [38]. Clearly it is not possible for any single item to capture sufficient detail so as to delineate the appropriate clinical pathway that should be pursued. Its utility lies instead in the ability to differentiate between those patients who have CSDs in the well being that can further be explored and subsequently treated.

Another issue with the use of an overall QOL measure is that it may involve issues that are beyond the purview of the clinician, such as financial or legal issues. In the age of comprehensive, multi-disciplinary, patient-centered care, however, identifying such issues can improve the efficacy of clinical care [39]. Indeed, much has been written about how issues beyond clinical care can impede or block positive clinical outcomes [40,41].

The overall LASA is routinely supplemented in clinical trials and practice by a series of other items relating to physical mental, emotional and spiritual well-being. These data are described elsewhere in the context of individual studies. The purpose of presenting only the overall item for this analysis is based on its universality and its demonstrated linkage to survival in a wide variety of patient populations.

## Conclusions

The present study indicates that the single-item measure of overall QOL has acceptable content and construct validity to be used as a clinical indicator of patient well-being. The relative capability for single items versus multiple item PRO measures to help us understand patient well-being is the focus of an R01-funded investigation presently ongoing. This project will compare psychometric properties, including the prognostic capability for survival, among the simple LASA measures, the PRO version of the Common Toxicity Criteria (PRO-CTCAE), and the PROMIS. This and other studies will further enhance our understanding of how we may “Cross-walk” results from alternative measures of the patient experience. Ultimately, this work will lead to a day when PROs are routinely incorporated into clinical care as a supplementary vital sign.

## Additional files

Additional file 1:
**LASA QOL Assessment scale used in studies.**
Additional file 2:
**Study Details.**
Additional file 3:
**Separate analyses of association of age category to LASA scores in observational (A) vs. cancer treatment trials (B).**
Additional file 4:
**Separate analyses of association of age category to LASA scores in observational (A) vs. cancer treatment trials (B).**

## Abbreviations

LASA: Linear analogue self assessment
CMD: Clinically meaningful difference
QOL: Quality of life
PROs: Patient-reported outcomes
PROMIS: Patient-reported outcome measurement information system
NCI: National Cancer Institute
PRO-CTCAE: PRO version of the common toxicity criteria
