# Supplementary material for: Normative data and clinically significant effect sizes for single-item numerical linear analogue self-assessment (LASA) scales
Source: Health Qual Life Outcomes. 2014 Dec 18;12:187. doi: 10.1186/s12955-014-0187-z (PMC4302440; doi:10.1186/s12955-014-0187-z)
Supplement: Additional file 1: — LASA QOL Assessment scale used in studies. [file 12955_2014_187_MOESM1_ESM.docx]

**Additional file 1.** LASA QOL Assessment scale used in studies
