# Supplementary material for: Normative data and clinically significant effect sizes for single-item numerical linear analogue self-assessment (LASA) scales
Source: Health Qual Life Outcomes. 2014 Dec 18;12:187. doi: 10.1186/s12955-014-0187-z (PMC4302440; doi:10.1186/s12955-014-0187-z)
Supplement: Additional file 2: — Study Details. [file 12955_2014_187_MOESM2_ESM.doc]

**Additional file** 2: Study Details

| **Study No.** | **Description** | **Tumor Site(s)** | **Assessments** | **Accrual** |
| --- | --- | --- | --- | --- |
| **952053**, | Pilot study of high-dose thoracic radiation therapy with concomitant cisplatin/etoposide in limited-stage small cell lung cancer | Lung | UNISCALE | 82 |
| **954651** | Phase II trial of oral 776C85 and oral 5-FU in untreated patients with unresectable or metastatic colorectal cancer | GI | UNISCALE | 79 |
| **959204** | Longitudinal descriptive study of quality of life in hospice patients and their caregivers | Psychosocial  Hospice/observational | POMS, UNISCALE | 58 |
| **959257** | Short-term chemoprevention trial in men with prostatic intraepithelial neoplasia using flutamide as and androgen deprivation agent | GI | POMS | 63 |
| **962451** | Phase II study of LU 103793 for treating advanced non-small cell lung cancer | Lung | UNISCALE | 17 |
| **969256** | Phase III double-blind study of glutamine vs. placebo for preventing acute diarrhea in patients receiving pelvic radiation therapy | GI, GU, Gyn, Other | UNISCALE | 129 |
| **971151** | Phase III evaluation of Benefin shark cartilage in patients with advanced cancer | Breast, GI | LASA, SDS, UNISCALE | 88 |
| **972451** | Phase III randomized, double-blind study of CAI and placebo in patients with advanced non-small cell lung cancer | Lung | FACT-G, UNISCALE | 17 |
| **952452** | A PH-2 Trial of Edatrexate in Combo w/ Vinblastine, Adriamycin, Cisplatin & Filgrastim in Pts w/ Advanced NSCLC | Lung | UNISCALE, FACT-L | 34 |
| **979202*** | Phase II clinical trial of dehydroepiandrosterone and Biaxin in monoclonal gammopathy of undetermined and borderline significance | Other | UNISCALE | 36 |
| **979251** | Phase III trial of evaluating low molecular weight heparin in patients with advanced cancer | Breast, GI, GU, Lung | LASA, SDS, UNISCALE | 141 |
| **979253** | Phase III randomized double-blind study of erythropoietin vs. placebo in anemic patients with cancer undergoing chemotherapy | Breast, GI, GU, Gyn, Head Neck , Hematologic, Lung, Lymphatic, Multiple, Musculoskeletal Sites, Other, Skin, Unknown | FACT-G, SDS, UNISCALE | 344 |
| **982452** | Phase II randomized study of docetaxel and gemcitabine for stage IIIB/IV non-small cell lung cancer | Lung | UNISCALE | 106 |
| **987251**,, | Phase II trial of pre-irradiation chemotherapy with BCNU, cisplatin and oral etoposide combined with radiation therapy in the treatment of grade 3 astrocytoma (anaplastic astrocytoma) | Neuro | FACT-G, LASA, POMS, SDS | 29 |
| **987252**, | Phase II trial of pre-irradiation chemotherapy with BCNU, cisplatin and oral etoposide combined with radiation therapy in the treatment of grade 4 astrocytoma (glioblastoma) | Neuro | FACT-G, LASA, POMS, SDS | 93 |
| **989251** | Phase II topical immunomodulatory therapy with imiquimod for the chemoprevention of recurrent and high-grade cervical intraepithelial neoplasia (CIN) | Gyn | UNISCALE | 57 |
| **MC0115** | Quality of life assessment of patients and caregivers participating in phase I clinical trials | GI, GU, Lung, Multiple, Musculoskeletal Sites | LASA | 46 |
| **MC0145** | Esophageal adenocarcinoma and Barrett’s esophagus registry | Breast, GI, GU, Head and Neck, Lung, Multiple, Unknown | LASA | 6017 |
| **MC997C** | Structured multidisciplinary intervention to improve quality of life in patients with advanced stage cancer | Breast, GI, GU, Gyn, Head and Neck, Lung, Multiple, Musculoskeletal Sites, Neuro, Skin, Unknown | LASA, POMS, SDS | 115 |
| **MC9991** | Pilot Study of assessing social support among cancer patients enrolled onto Mayo Clinic comprehensive cancer center clinical trials: a comparison of younger versus older adults | Breast, GI, GU, Gyn, Head and Neck, Lung, Other, Unknown | SDS, UNISCALE | 51 |
| **MC99C2** | Phase III randomized, double-blind, placebo-controlled crossover trial of glutamine in preventing paclitaxel – associated myalgias and arthralgias | Breast, GI, GU, Gyn, Lung, Multiple | LASA, SDS | 36 |
| **N0021** | Phase II study of gemcitabine and epirubicin for the treatment of mesothelioma | Head and Neck | SDS | 69 |
| **N0022** | Oral vinorelbine for the treatment of metastatic non-small cell lung cancer in patients >=65 years of age: a phase II trial of efficacy, toxicity, and patients’ perceived preference for oral therapy | Lung | UNISCALE | 59 |
| **N0044**, , , | Phase II trial of preoperative radiation and chemotherapy (paclitaxel, carboplatin, and continuous infusion 5-FU) for locally advanced esophageal cancer | GI | SDS, UNISCALE | 56 |
| **N0048*** | Phase II studies of CPT-11 or 5-FU/CF in patients with metastatic colorectal carcinoma previously treated with OXAL or a combination of CPT-11 and OXAL | GI | SDS, UNISCALE | 19 |
| **N0074** | Phase II study of ZD 1839 in newly diagnosed patients with glioblastoma (grade 4 astrocytoma) | Neuro | FACT-G, LASA, POMS, SDS | 98 |
| **N014C**, | Randomized phase II trial of PS-341 and gemcitabine in patients with metastatic pancreatic adenocarcinoma | GI | FACT-G, SDS, UNISCALE | 90 |
| **N01C4** | Phase III double-blind, placebo-controlled randomized comparison of Zinc Sulfate versus placebo for the prevention of altered taste in patients with head and neck cancer during radiation | Head and neck | LASA | 173 |
| **N0242** | Phase II study of docetaxel and capecitabine in patients with measurable metastatic adenocarcinoma of the stomach and gastro esophageal junction | GI | LASA | 46 |
| **N0272*** | Phase I/II trial of Imatinib mesylate; (Gleevec; STI-571) in treatment of recurrent oligodendroglioma and mixed oligoastrocytoma | Neuro | LASA | 64 |
| **N9741**, , | Randomized Phase III trial of combinations of OXAL, 5-FU, and CPT-11 as initial treatment of patients with advanced adenocarcinoma of the colon and rectum | GI | SDS, UNISCALE | 1751 |
| **N9841** | Randomized Phase III equivalence trial of CPT-11 versus OXAL/5-FU/.CF in patients with advanced colorectal carcinoma previously treated with 5-FU | GI | SDS, UNISCALE | 507 |
| **N9923** | Phase I-II study of topotecan and paclitaxel followed by high-dose thoracic radiation therapy with concomitant cisplatin/etoposide and amifostine in limited-stage small cell lung cancer | Lung | UNISCALE | 34 |
| **N9946** | Phase II study of OXAL, 5-FU and CF in patients with metastatic colorectal carcinoma previously treated with CPT-11 | GI | SDS, UNISCALE | 48 |
| **N99C7**, | Phase III comparison of depomedroxyprogesterone acetate (DPROV) to venlafaxine for managing hot flashes | Breast | UNISCALE | 227 |
| **Mayo (INT 0123)** | Mayo Epidemiology and Genetics of Lung Cancer Research Program (EGLC) | Lung | LASA | 529 |
| Mayo (INT 0124) | Mayo Epidemiology and Genetics of Lung Cancer Research Program (EGLC) | Lung | LASA | 1409 |
| Survey data | Healthy NCCTG Volunteers |  | LASA | 54 |
| 934653(INT 0146) | Lap – Colon Pts-Pre Surgery  Lap – Colon Pts 2wks Post Surgery | Colon | LASA | 870 |
| 959204, | Hospice Caregivers/patients |  | LASA | 58 |
| Survey Data, | Mayo Physicians^82, 83^ |  | LASA | 460 |
| QOL_019, , | Mayo Residents/Students |  | LASA | 295 |

*, no related publications at this time

**References**

1. Schild SE, Bonner JA, Hillman S, Kozelsky TF, Vigliotti AP, Marks RS, Graham DL, Soori GS, Kugler JW, Tenglin RC, et al: **Results of a phase II study of high-dose thoracic radiation therapy with concurrent cisplatin and etoposide in limited-stage small-cell lung cancer (NCCTG 95-20-53).** *Journal of clinical oncology : official journal of the American Society of Clinical Oncology* 2007, **25:**3124-3129.

2. Huschka MM, Mandrekar SJ, Schaefer PL, Jett JR, Sloan JA: **A pooled analysis of quality of life measures and adverse events data in north central cancer treatment group lung cancer clinical trials.** *Cancer* 2007, **109:**787-795.

3. Hobday TJ, Kugler JW, Mahoney MR, Sargent DJ, Sloan JA, Fitch TR, Krook JE, O'Connell MJ, Mailliard JA, Tirona MT, et al: **Efficacy and quality-of-life data are related in a phase II trial of oral chemotherapy in previously untreated patients with metastatic colorectal carcinoma.** *Journal of clinical oncology : official journal of the American Society of Clinical Oncology* 2002, **20:**4574-4580.

4. Gill P, Kaur JS, Rummans T, Novotny PJ, Sloan JA: **The hospice patient's primary caregiver. What is their quality of life?** *Journal of psychosomatic research* 2003, **55:**445-451.

5. Alberts SR, Novotny PJ, Sloan JA, Danella J, Bostwick DG, Sebo TJ, Blute ML, Fitch TR, Levitt R, Lieberman R, Loprinzi CL: **Flutamide in men with prostatic intraepithelial neoplasia: a randomized, placebo-controlled chemoprevention trial.** *American journal of therapeutics* 2006, **13:**291-297.

6. Marks RS, Graham DL, Sloan JA, Hillman S, Fishkoff S, Krook JE, Okuno SH, Mailliard JA, Fitch TR, Addo F: **A phase II study of the dolastatin 15 analogue LU 103793 in the treatment of advanced non-small-cell lung cancer.** *American journal of clinical oncology* 2003, **26:**336-337.

7. Kozelsky TF, Meyers GE, Sloan JA, Shanahan TG, Dick SJ, Moore RL, Engeler GP, Frank AR, McKone TK, Urias RE, et al: **Phase III double-blind study of glutamine versus placebo for the prevention of acute diarrhea in patients receiving pelvic radiation therapy.** *Journal of clinical oncology : official journal of the American Society of Clinical Oncology* 2003, **21:**1669-1674.

8. Loprinzi CL, Levitt R, Barton DL, Sloan JA, Atherton PJ, Smith DJ, Dakhil SR, Moore DF, Jr., Krook JE, Rowland KM, Jr., et al: **Evaluation of shark cartilage in patients with advanced cancer: a North Central Cancer Treatment Group trial.** *Cancer* 2005, **104:**176-182.

9. Johnson EA, Marks RS, Mandrekar SJ, Hillman SL, Hauge MD, Bauman MD, Wos EJ, Moore DF, Kugler JW, Windschitl HE, et al: **Phase III randomized, double-blind study of maintenance CAI or placebo in patients with advanced non-small cell lung cancer (NSCLC) after completion of initial therapy (NCCTG 97-24-51).** *Lung Cancer* 2008, **60:**200-207.

10. Colon-Otero G, Niedringhaus RD, Hillman SH, Geyer S, Sloan J, Krook JE, Windschitl HE, Marks RS, Wiesenfeld M, Tschetter LK, Jett JJ: **A phase II trial of edatrexate, vinblastine, adriamycin, cisplastin, and filgrastim (EVAC/G-CSF) in patients with non-small-cell carcinoma of the lungs: a North Central Cancer Treatment Group Trial.** *American journal of clinical oncology* 2001, **24:**551-555.

11. Sideras K, Schaefer PL, Okuno SH, Sloan JA, Kutteh L, Fitch TR, Dakhil SR, Levitt R, Alberts SR, Morton RF, et al: **Low-molecular-weight heparin in patients with advanced cancer: a phase 3 clinical trial.** *Mayo Clinic proceedings* 2006, **81:**758-767.

12. Witzig TE, Silberstein PT, Loprinzi CL, Sloan JA, Novotny PJ, Mailliard JA, Rowland KM, Alberts SR, Krook JE, Levitt R, Morton RF: **Phase III, randomized, double-blind study of epoetin alfa compared with placebo in anemic patients receiving chemotherapy.** *Journal of clinical oncology : official journal of the American Society of Clinical Oncology* 2005, **23:**2606-2617.

13. Jatoi A, Hillman S, Stella P, Green E, Adjei A, Nair S, Perez E, Amin B, Schild SE, Castillo R, Jett JR: **Should elderly non-small-cell lung cancer patients be offered elderly-specific trials? Results of a pooled analysis from the North Central Cancer Treatment Group.** *Journal of clinical oncology : official journal of the American Society of Clinical Oncology* 2005, **23:**9113-9119.

14. Locke DE, Decker PA, Sloan JA, Brown PD, Malec JF, Clark MM, Rummans TA, Ballman KV, Schaefer PL, Buckner JC: **Validation of single-item linear analog scale assessment of quality of life in neuro-oncology patients.** *Journal of pain and symptom management* 2007, **34:**628-638.

15. Rao RD, Krishnan S, Fitch TR, Schomberg PJ, Dinapoli RP, Nordstrom K, Scheithauer B, O'Fallon JR, Maurer MJ, Buckner JC: **Phase II trial of carmustine, cisplatin, and oral etoposide chemotherapy before radiotherapy for grade 3 astrocytoma (anaplastic astrocytoma): results of North Central Cancer Treatment Group trial 98-72-51.** *International journal of radiation oncology, biology, physics* 2005, **61:**380-386.

16. Brown PD, Maurer MJ, Rummans TA, Pollock BE, Ballman KV, Sloan JA, Boeve BF, Arusell RM, Clark MM, Buckner JC: **A prospective study of quality of life in adults with newly diagnosed high-grade gliomas: the impact of the extent of resection on quality of life and survival.** *Neurosurgery* 2005, **57:**495-504; discussion 495-504.

17. Pachman DR, Barton DL, Clayton AC, McGovern RM, Jefferies JA, Novotny PJ, Sloan JA, Loprinzi CL, Gostout BS: **Randomized clinical trial of imiquimod: an adjunct to treating cervical dysplasia.** *American journal of obstetrics and gynecology* 2012, **206:**42 e41-47.

18. Sloan J, Loprinzi CL, O'Fallon JR, Kuross SA, Miser AW, Mahoney MR, Heid IM: **A randomized comparison of four quality of life (qol) tools in patients with advanced cancer.** *Proc Am Soc Clin Oncol* 1996, **15:**508.

19. Prasad GA, Buttar NS, Wongkeesong LM, Lewis JT, Sanderson SO, Lutzke LS, Borkenhagen LS, Wang KK: **Significance of neoplastic involvement of margins obtained by endoscopic mucosal resection in Barrett's esophagus.** *The American journal of gastroenterology* 2007, **102:**2380-2386.

20. Rummans TA, Clark MM, Sloan JA, Frost MH, Bostwick JM, Atherton PJ, Johnson ME, Gamble G, Richardson J, Brown P, et al: **Impacting quality of life for patients with advanced cancer with a structured multidisciplinary intervention: a randomized controlled trial.** *Journal of clinical oncology : official journal of the American Society of Clinical Oncology* 2006, **24:**635-642.

21. Novotny P, Sloan J, Guse L, Alberts SR, Golberg R, Gregory D, Hartmann L, Johnson M, Rummans T: **A pilot study assessing social support among cancer patients enrolled on clinical trials: a comparison of younger versus older adults.** *Proc Am Soc Clin Oncol* 2003, **22 (abstract 2992)**.

22. Jacobson SD, Loprinzi CL, Sloan JA, Wilke JL, Novotny PJ, Okuno SH, Jatoi A, Moynihan TJ: **Glutamine does not prevent paclitaxel-associated myalgias and arthralgias.** *The journal of supportive oncology* 2003, **1:**274-278.

23. Okuno SH, Delaune R, Sloan JA, Foster NR, Maurer MJ, Aubry MC, Rowland KM, Jr., Soori GS, Nikcevich DA, Kardinal CG, et al: **A phase 2 study of gemcitabine and epirubicin for the treatment of pleural mesothelioma: a North Central Cancer Treatment Study, N0021.** *Cancer* 2008, **112:**1772-1779.

24. Kanard A, Jatoi A, Castillo R, Geyer S, Schulz TK, Fitch TR, Rowland KM, Nair S, Krook JE, Kugler JW: **Oral vinorelbine for the treatment of metastatic non-small cell lung cancer in elderly patients: a phase II trial of efficacy and toxicity.** *Lung Cancer* 2004, **43:**345-353.

25. Jatoi A: **Aggressive multimodality therapy for patients with locally advanced esophageal cancer: is there a role for amifostine?** *Seminars in oncology* 2003, **30:**72-75.

26. Jatoi A, Martenson J, Mahoney MR, Lair BS, Brindle JS, Nichols F, Caron N, Rowland K, Tschetter L, Alberts S: **Results of a planned interim toxicity analysis with trimodality therapy, including carboplatin AUC = 4, paclitaxel, 5-fluorouracil, amifostine, and radiation for locally advanced esophageal cancer: preliminary analyses and treatment recommendations from the North Central Cancer Treatment Group.** *International seminars in surgical oncology : ISSO* 2004, **1:**9.

27. Jatoi A, Martenson JA, Foster NR, McLeod HL, Lair BS, Nichols F, Tschetter LK, Moore DF, Jr., Fitch TR, Alberts SR: **Paclitaxel, carboplatin, 5-fluorouracil, and radiation for locally advanced esophageal cancer: phase II results of preliminary pharmacologic and molecular efforts to mitigate toxicity and predict outcomes: North Central Cancer Treatment Group (N0044).** *American journal of clinical oncology* 2007, **30:**507-513.

28. Jatoi A, Thomas CR, Jr.: **Esophageal cancer and the esophagus: challenges and potential strategies for selective cytoprotection of the tumor-bearing organ during cancer treatment.** *Seminars in radiation oncology* 2002, **12:**62-67.

29. Alberts SR, Foster NR, Morton RF, Kugler J, Schaefer P, Wiesenfeld M, Fitch TR, Steen P, Kim GP, Gill S: **PS-341 and gemcitabine in patients with metastatic pancreatic adenocarcinoma: a North Central Cancer Treatment Group (NCCTG) randomized phase II study.** *Annals of oncology : official journal of the European Society for Medical Oncology / ESMO* 2005, **16:**1654-1661.

30. Jatoi A, Alberts SR, Foster N, Morton R, Burch P, Block M, Nguyen PL, Kugler J: **Is bortezomib, a proteasome inhibitor, effective in treating cancer-associated weight loss? Preliminary results from the North Central Cancer Treatment Group.** *Supportive care in cancer : official journal of the Multinational Association of Supportive Care in Cancer* 2005, **13:**381-386.

31. Halyard MY, Jatoi A, Sloan JA, Bearden JD, 3rd, Vora SA, Atherton PJ, Perez EA, Soori G, Zalduendo AC, Zhu A, et al: **Does zinc sulfate prevent therapy-induced taste alterations in head and neck cancer patients? Results of phase III double-blind, placebo-controlled trial from the North Central Cancer Treatment Group (N01C4).** *International journal of radiation oncology, biology, physics* 2007, **67:**1318-1322.

32. Giordano KF, Jatoi A, Stella PJ, Foster N, Tschetter LK, Alberts SR, Dakhil SR, Mailliard JA, Flynn PJ, Nikcevich DA: **Docetaxel and capecitabine in patients with metastatic adenocarcinoma of the stomach and gastroesophageal junction: a phase II study from the North Central Cancer Treatment Group.** *Annals of oncology : official journal of the European Society for Medical Oncology / ESMO* 2006, **17:**652-656.

33. Erlichman C, Goldberg RM, O'Connell MJ: **Irinotecan plus fluorouracil and leucovorin for metastatic colorectal cancer.** *The New England journal of medicine* 2001, **344:**305; author reply 306-307.

34. Sargent DJ, Niedzwiecki D, O'Connell MJ, Schilsky RL: **Recommendation for caution with irinotecan, fluorouracil, and leucovorin for colorectal cancer.** *The New England journal of medicine* 2001, **345:**144-145; author reply 146.

35. Goldberg RM: **N9741: a phase III study comparing irinotecan to oxaliplatin-containing regimens in advanced colorectal cancer.** *Clinical colorectal cancer* 2002, **2:**81.

36. Kim GP, Sargent DJ, Mahoney MR, Rowland KM, Jr., Philip PA, Mitchell E, Mathews AP, Fitch TR, Goldberg RM, Alberts SR, Pitot HC: **Phase III noninferiority trial comparing irinotecan with oxaliplatin, fluorouracil, and leucovorin in patients with advanced colorectal carcinoma previously treated with fluorouracil: N9841.** *Journal of clinical oncology : official journal of the American Society of Clinical Oncology* 2009, **27:**2848-2854.

37. Garces YI, Okuno SH, Schild SE, Mandrekar SJ, Bot BM, Martens JM, Wender DB, Soori GS, Moore DF, Jr., Kozelsky TF, Jett JR: **Phase I North Central Cancer Treatment Group Trial-N9923 of escalating doses of twice-daily thoracic radiation therapy with amifostine and with alternating chemotherapy in limited stage small-cell lung cancer.** *International journal of radiation oncology, biology, physics* 2007, **67:**995-1001.

38. Pitot HC, Wiesenfeld M, Mahoney MR, Alberts SR, Schaefer PL, Nair SC, Mailliard JA, Rowland KM, Kugler JW, Goldberg RM: **A phase II trial of oxaliplatin (oxal), 5-fluorouracil (5FU), and leucovorin (lv) in patients (pts) with metastatic colon cancer (m-cc) refractory to irinotecan (cpt11) based therapy: a North Central Cancer Treatment Group (NCCTG) study.** *Proc Am Soc Clin Oncol* 2003, **22:**261.

39. Barton D, Loprinzi C, Quella S, Sloan J, Pruthi S, Novotny P: **Depomedroxyprogesterone acetate for hot flashes.** *Journal of pain and symptom management* 2002, **24:**603-607.

40. Loprinzi CL, Levitt R, Barton D, Sloan JA, Dakhil SR, Nikcevich DA, Bearden JD, 3rd, Mailliard JA, Tschetter LK, Fitch TR, Kugler JW: **Phase III comparison of depomedroxyprogesterone acetate to venlafaxine for managing hot flashes: North Central Cancer Treatment Group Trial N99C7.** *Journal of clinical oncology : official journal of the American Society of Clinical Oncology* 2006, **24:**1409-1414.

41. Sloan JA, Zhao X, Novotny PJ, Wampfler J, Garces Y, Clark MM, Yang P: **Relationship between deficits in overall quality of life and non-small-cell lung cancer survival.** *Journal of clinical oncology : official journal of the American Society of Clinical Oncology* 2012, **30:**1498-1504.

42. Stucky CH, Pockaj BA, Novotny P, Sloan JA, Sargent DJ, O'Connell MJ, Beart RW, Skibber J, Nelson H, Weeks JC: **Individual item analysis of quality of life assessments related to laparoscopic-assisted colectomy in hte COST Trial 93-46-53 (INT 0146).** *Ann Surg Oncol* 2010, **Suppl 1:**S36.

43. Bretscher M, Rummans T, Sloan J, Kaur J, Bartlett A, Borkenhagen L, Loprinzi C: **Quality of life in hospice patients. A pilot study.** *Psychosomatics* 1999, **40:**309-313.

44. Shanafelt TD, West CP, Sloan JA, Novotny PJ, Poland GA, Menaker R, Rummans TA, Dyrbye LN: **Career fit and burnout among academic faculty.** *Archives of internal medicine* 2009, **169:**990-995.

45. West CP, Tan AD, Habermann TM, Sloan JA, Shanafelt TD: **Association of resident fatigue and distress with perceived medical errors.** *JAMA* 2009, **302:**1294-1300.

46. Shanafelt TD, West C, Zhao X, Novotny P, Kolars J, Habermann T, Sloan J: **Relationship between increased personal well-being and enhanced empathy among internal medicine residents.** *Journal of general internal medicine* 2005, **20:**559-564.

47. West CP, Huschka MM, Novotny PJ, Sloan JA, Kolars JC, Habermann TM, Shanafelt TD: **Association of perceived medical errors with resident distress and empathy: a prospective longitudinal study.** *JAMA* 2006, **296:**1071-1078.
