# Supplementary material for: Normative data and clinically significant effect sizes for single-item numerical linear analogue self-assessment (LASA) scales
Source: Health Qual Life Outcomes. 2014 Dec 18;12:187. doi: 10.1186/s12955-014-0187-z (PMC4302440; doi:10.1186/s12955-014-0187-z)
Supplement: Additional file 3: — Separate analyses of association of age category to LASA scores in observational (A) vs. cancer treatment trials (B). [file 12955_2014_187_MOESM3_ESM.doc]

**Additional file 3. Separate analyses of association of age category to LASA scores in observational (A) vs. cancer treatment trials (B)**

1. Observational Studies

| **Overall QOL by Age Category for Observational Studies** | | | | | | | |
| --- | --- | --- | --- | --- | --- | --- | --- |
|  | Missing (N=1359) | <50 (N=17) | 50-64 (N=41) | 65-71 (N=37) | 72+ (N=63) | Total (N=1517) | p value |
| **Overall QOL** |  |  |  |  |  |  | 0.1225^1^ |
| N | 1352 | 15 | 40 | 31 | 63 | 149 |  |
| Mean (SD) | 7.1 (1.8) | 5.7 (1.8) | 6.6 (2.0) | 7.2 (2.0) | 6.6 (1.8) | 6.7 (1.9) |  |
| Median | 7.0 | 5.0 | 6.6 | 7.5 | 6.5 | 6.8 |  |
| Q1, Q3 | 6.0, 8.0 | 4.3, 7.5 | 5.3, 7.8 | 5.0, 8.8 | 5.0, 7.5 | 5.0, 8.0 |  |
| Range | (1.0-10.0) | (2.5-8.3) | (2.0-9.8) | (3.5-9.8) | (2.5-10.0) | (2.0-10.0) |  |
| ^1^Kruskal Wallis | | | | | | | |

B. Cancer treatment studies

| **Overall QOL by Age Category for Cancer Treatment Studies** | | | | | | | |
| --- | --- | --- | --- | --- | --- | --- | --- |
|  | Missing (N=1539) | <50 (N=893) | 50-64 (N=2337) | 65-71 (N=1407) | 72+ (N=1602) | Total (N=7778) | p value |
| **Overall QOL** |  |  |  |  |  |  | 0.0056^1^ |
| N | 1532 | 886 | 2310 | 1379 | 1553 | 6128 |  |
| Mean (SD) | 7.1 (2.4) | 7.6 (1.9) | 7.7 (1.9) | 7.6 (2.0) | 7.4 (2.1) | 7.6 (2.0) |  |
| Median | 7.7 | 8.0 | 8.0 | 8.0 | 8.0 | 8.0 |  |
| Q1, Q3 | 5.3, 9.0 | 6.7, 9.0 | 6.8, 9.1 | 6.4, 9.1 | 6.0, 9.0 | 6.5, 9.0 |  |
| Range | (0.0-10.0) | (1.1-10.0) | (0.0-10.0) | (0.0-10.0) | (0.0-10.0) | (0.0-10.0) |  |
| ^1^Kruskal Wallis | | | | | | | |
