# Supplementary material for: Normative data and clinically significant effect sizes for single-item numerical linear analogue self-assessment (LASA) scales
Source: Health Qual Life Outcomes. 2014 Dec 18;12:187. doi: 10.1186/s12955-014-0187-z (PMC4302440; doi:10.1186/s12955-014-0187-z)
Supplement: Additional file 4: — Separate analyses of association of age category to LASA scores in observational (A) vs. cancer treatment trials (B). [file 12955_2014_187_MOESM4_ESM.doc]

**Additional file 4. Separate analyses of association of age category to LASA scores in observational (A) vs. cancer treatment trials (B)**

1. Observational Studies

| **Overall QOL by Gender for Observational Studies** | | | | | |
| --- | --- | --- | --- | --- | --- |
|  | Missing (N=1391) | F (N=57) | M (N=69) | Total (N=1517) | p value |
| **Overall QOL** |  |  |  |  | 0.0276^1^ |
| N | 1377 | 56 | 68 | 124 |  |
| Mean (SD) | 7.1 (1.8) | 7.0 (2.1) | 6.3 (1.7) | 6.6 (1.9) |  |
| Median | 7.0 | 7.5 | 6.3 | 6.6 |  |
| Q1, Q3 | 6.0, 8.0 | 5.3, 8.8 | 5.0, 7.5 | 5.0, 7.9 |  |
| Range | (1.0-10.0) | (2.0-9.8) | (3.5-9.8) | (2.0-9.8) |  |
| ^1^Kruskal Wallis | | | | | |

B. Cancer treatment studies

| **Overall QOL by Gender for Cancer Treatment Studies** | | | | | |
| --- | --- | --- | --- | --- | --- |
|  | Missing (N=1540) | F (N=2778) | M (N=3460) | Total (N=7778) | p value |
| **Overall QOL** |  |  |  |  | 0.4201^1^ |
| N | 1529 | 2716 | 3415 | 6131 |  |
| Mean (SD) | 7.1 (2.4) | 7.6 (1.9) | 7.5 (2.0) | 7.6 (2.0) |  |
| Median | 7.7 | 8.0 | 8.0 | 8.0 |  |
| Q1, Q3 | 5.3, 9.0 | 6.7, 9.0 | 6.2, 9.1 | 6.5, 9.0 |  |
| Range | (0.0-10.0) | (0.0-10.0) | (0.0-10.0) | (0.0-10.0) |  |
| ^1^Kruskal Wallis | | | | | |
